# L-Aminoacid Oxidase from *Bothrops leucurus* Venom Induces Nephrotoxicity via Apoptosis and Necrosis

**DOI:** 10.1371/journal.pone.0132569

**Published:** 2015-07-20

**Authors:** Isabel C. O. Morais, Gustavo J. S. Pereira, M. Orzáez, Roberta J. B. Jorge, Claudia Bincoletto, Marcos H. Toyama, Helena S. A. Monteiro, Soraya S. Smaili, Enrique Pérez-Payá, Alice M. C. Martins

**Affiliations:** 1 Department of Physiology and Pharmacology, Faculty of Medicine, Federal University of Ceará, Fortaleza, Ceará, Brazil; 2 Department of Pharmacology, Federal University of São Paulo (UNIFESP), São Paulo, Brazil; 3 Department of Medicinal Chemistry, Centro de Investigación Príncipe Felipe, Valencia, Spain; 4 São Vicente Unit, Paulista Coastal Campus, São Paulo State University (UNESP), São Paulo, Brazil; 5 Department of Clinical and Toxicological Analysis, Federal University of Ceará, Fortaleza, Ceará, Brazil; Charité Universitätsmedizin Berlin, GERMANY

## Abstract

Acute renal failure is a common complication caused by *Bothrops* viper envenomation. In this study, the nefrotoxicity of a main component of *B*. *leucurus* venom called L-aminoacid oxidase (LAAO-*Bl*) was evaluated by using tubular epithelial cell lines MDCK and HK-2 and perfused kidney from rats. LAAO-*Bl* exhibited cytotoxicity, inducing apoptosis and necrosis in MDCK and HK-2 cell lines in a concentration-dependent manner. MDCK apoptosis induction was accompanied by Ca^2+^ release from the endoplasmic reticulum, reactive oxygen species (ROS) generation and mitochondrial dysfunction with enhanced expression of Bax protein levels. LAAO-*Bl* induced caspase-3 and caspase-7 activation in both cell lines. LAAO-*Bl* (10 μg/mL) exerts significant effects on the isolated kidney perfusion increasing perfusion pressure and urinary flow and decreasing the glomerular filtration rate and sodium, potassium and chloride tubular transport. Taken together our results suggest that LAAO-*Bl* is responsible for the nephrotoxicity observed in the envenomation by snakebites. Moreover, the cytotoxic of LAAO-*Bl* to renal epithelial cells might be responsible, at least in part, for the nephrotoxicity observed in isolated kidney.

## Introduction

Envenomation by snakebites, recognized as a neglected tropical disease (NDT) by the World Health Organization (WHO), constitutes an important worldwide public health problem, particularly in the rural areas of tropical countries as Africa, Asia and Latin America. In Brazil, most accidents with species notification are due to vipers of the *Bothrops* genus (83.8%) [1, 2].

*Bothrops* venoms are especially interesting as they contain a complex mixture of proteins and peptides, with a wide range of biological and pharmacological activities. More than 90% of their dry weight consists of proteins that are capable of interfering with a variety of physiological processes [3, 4].

An important protein superfamily present in all snake venoms is L-amino acid oxidase (LAAO), a flavoenzyme that catalyzes oxidative deamination of L-amino acids to synthetize the corresponding α-ketoacid, hydrogen peroxide (H_2_O_2_) and ammonia. LAAOs are present in most snake venoms, with approximately 30% of the total protein content and contributes to venom toxicity upon envenomation [5, 6].

LAAOs have been studied for the last decade and have attracted the interest of researchers due to their polyfunctional effects on different biological systems. LAAO has been recognized as a multifunctional enzyme with evident biological effects on platelet aggregation, edema formation, hemorrhage, antimicrobial, antiviral, anti-HIV action and is cytotoxic to tumor cells. Although most of LAAOs biological effects might be mediated by H_2_O_2_, the mechanism of their actions is still unclear [7].

The pit viper *Bothrops leucurus* (White-tailed-jararaca) is a poisonous snake whose natural habitat is the northeast of Brazil. The biological effects due to envenomation have a similar profile to those observed with other *Bothrops* snakes, such as coagulant, hemorrhagic and fibrinolytic activity, as well as renal failure *in vivo* [8, 2].

Recently, we observed that *Bothrops leucurus* venom induces nephrotoxicity in isolated perfused kidneys of rats, associated with cytotoxicity against renal tubular epithelial cells [2]. Here, we extended these studies by evaluating the precise role of a major component of *B*. *leucurus* venom, LAAO, to better understand the biological effects produced by envenomation. In order to clarify LAAO nephrotoxicity, we investigated not only its nephrotoxic potential, but also its cytotoxicity against renal epithelial cell lines.

## Materials and Methods

### Venom, chemicals and drugs

LAAO from *B*. *leucurus* venom was isolated and purified as described by Torres *et al*. (2010) [9]. DMEM medium, phenylhydrazone (FCCP), 3-(4, 5-dimethylthiazolyl-2)-2,5-diphenyltetrazolium bromide (MTT) and catalase kit were purchased from Sigma Chemical Co. (St. Louis, MO, USA). Tetramethylrhodamine ethyl ester (TMRE), Fura-2AM and 2,7dichlorodihydrofluorescein diacetate (DCFH-DA) were purchased from Molecular Probes (Eugene, OR, USA). AnnexinV/FITC Apoptosis Detection Kit was from BD Pharmigen (CA, USA). Z-Val-Ala-Asp (OMe)-fluoromethylketone (zVAD), catalase and BAPTA-AM were from Tocris (Bristol, UK).

### Cell culture

Human kidney (HK-2) and Canine kidney (MDCK) cell lines were cultured in Dulbecco’s modified Eagle’s medium (DMEM, Sigma-Aldrich) supplemented with 10% fetal bovine serum (FBS, Sigma-Aldrich), 2 mM L-glutamine, 1% penicillin/streptomycin solution at 37°C

### MTT mitochondrial dysfunction assay

Cell viability was also measured using a standard methyl thiazol tetrazolium (MTT) assay. Briefly, viable cells were plated into 96-well flat microplates (Corning, USA) in DMEM medium, treated with different concentrations of LAAO-B*l* and incubated at 37°C at 12 h. It was added 10 μg/well of MTT (5 mg/mL) and incubated for 4 h, when DMSO (Merck, Germany) was added to each well to solubilize the formazan. The cell viability measurements were accessed using 570 nm on Wallac 1420 workstation.

### Lactate Dehydrogenase (LDH) Release

Cytotoxicity induced by LAAO-*Bl* was assessed by lactate dehydrogenase (LDH) leakage into the culture medium. After 12 hours of treatment, cells supernatant was removed and reactive mixture was added for 30 min at room temperature under protection from light for determination LDH release using the Promega kit (6179A). Plates were read at 440 nm on Wallac 1420 workstation.

### Annexin V-FITC and propidium iodide (PI) staining

Cells treated with different concentrations of LAAO-*Bl* were stained with fluorescein isothiocyanate (FITC)-conjugated to annexinV/propidium iodide (PI) according to manufacturer’s instructions. The population of annexinV-PI viable cells and annexinV^+^ apoptotic cells was evaluated by flow-cytometry. Data were collected in a FACS Calibur (Becton-Kickinson, Mountain View, Calif.) and analyzed using Cell Quest software (Becton-Dickinson).

### Mitochondrial membrane potentiality (ΔΨm) measurements

#### Fluorescence Microscope

To evaluate the effects of LAAO-*Bl* (50 μg/mL) on **Δ**Ψm, cells were plated on coverslips and incubated with TMRE (25 nM, 15 min at 37°C), a potentiometric and cationic indicator dye that accumulates preferentially into the energized mitochondria. Before the addition of LAAO-*Bl*, cells were exposed to light for at least 5 min to ensure a stable baseline. The experiments were performed in the presence of the dye to diminish the decrease in fluorescence due to photo bleaching [10]. TMRE fluorescence (548 nm excitation and 585 nm emission) was acquired using a high resolution fluorescence microscope (Nikon TE 300, Nikon, Osaka, Japan) coupled to a CCD camera (CoolSnap-Roper Scientific Inc., Princeton Instruments, Princeton, NJ, USA). Images were acquired by BioIP software (Anderson Eng., USA).

#### Flow cytometry

MDCK cells were treated with LAAO-*Bl* (50 μg/mL, 12 h). After this period, the collected cells incubated with TMRE (25 nM, 15 min, 37°C) in the dark. The cells were analyzed using FL2 by flow cytometry.

### Ca^2+^ measurements

To evaluate the Ca^2+^ handling, MDCK cells were plated on coverslips after loaded with 3 μM of acetoxymethyl ester of fura-2 (Fura-2AM) in a regular buffer containing (mM): 130 NaCl, 5.36 KCl, 0.8 MgSO4, 1 Na_2_HPO_4_, 25 glucose, 20 HEPES, pH 7.3 for 30 min. Cytoplasmic Ca^2+^ measurements were evaluated by fluorescence microscopy (Nikon TE 300; Nikon, Osaka, Japan) coupled to a CCD camera (Quantix 512-Roper Scientific Inc., Princeton Instruments, Princeton, NJ). Images were acquired in BioIP software (Anderson Eng, Delaware, USA). Basal Ca^2+^ levels were considered the first 15 images, and then exposed to LAAO-*Bl* (50 μg/mL). Fura-2 fluorescence (emission = 510 nm) was monitored following alternate excitation at 340 and 380 nm. Percentages were expressed as ratio (340/380) values, normalized from the basal fluorescence and data were normalized by the (F − F_0_)/F_0_ × 100 formula, in which F_0_ represents the basal Ca^2+^ levels.

### Reactive oxygen species (ROS) measurements

Cytosolic ROS was measured using 2,7-dichlorodihydrofluorescein diacetate (DCFH-DA), which can readily enter cells and be cleaved by esterase to yield DCFH, a polar, nonfluorescent product. ROS in cells promotes the oxidation of DCFH to yield the fluorescent product, dichlorofluorescein. After treatment (LAAO-*Bl* 50 μg/mL, 12 h), cells were collected and then incubated in PBS containing the reagent DCFH-DA (5 μM, 30 min at 37°C). After that, submitted to flow cytometric analysis using a FACScan flow cytometer. Tert-Butyl hydroperoxide (*t*-BHP, 5 μM) was used as positive control. Data based on the FL1 channel were analyzed with the CellQuest program.

### Caspase 3/7 activity measurements

Extracts collected from LAAO- *Bl* (50 μg/mL, 12 h)-treated cells were lysed in extraction buffer (50 mM PIPES, 50 mM KCl, 5 mM EDTA, 2 mM MgCl_2_, 2 mM DTT, supplemented with protease inhibitors). After 30 min on ice, cell lysates were centrifuged at 13.2000 rpm for 10 min, then the supernatants collected were quantified using the BCA protein assay. Total protein (40 μg) was mixed with 200 μL of caspase assay buffer (PBS, 10% glycerol, 0.1 mM EDTA, 2 mM DTT) containing 20 μM of Ac-DEVD-afc (Enzo Life Sciences) of the caspase-3 substrate. DVDase activity was continuously monitored following the release of fluorescent afc at 37°C with Wallac 1420 Workstation (λ exc = 400 nm; λ em = 508 nm).

### Immunoblotting

Whole cell extracts were obtained by lysing cells in a buffer containing 25 mM Tris-HCl pH 7.4, 1 mM EDTA, 1 mM EGTA, 1% SDS, plus protease and phosphatase inhibitors. The protein concentration was determined by the BCA protein assay. Cell lysates were resolved by SDS-PAGE, transferred to nitrocellulose membranes, blocked with 5% non fat milk, washed with 0.1% Tween/PBS and incubated overnight with a specific primary antibody. Membranes were washed and probed with the appropriate secondary antibody conjugated to horseradish peroxidase (Amersham Pharmacia Biotech). The antibodies against Caspase 3 (#96615) and Caspase 7(#9492) were obtained from Cell Signaling, α-tubulin antibody (#T8203) was obtained from Sigma-Aldrich and Bax (Sc 493) was obtained from Santa Cruz Biotechnology.

### Kidney perfusion

Adult male Wistar rats (260–320 g) were fasted for 24 h with free access to water. The rats were anesthetized with sodium pentobarbitone (50 mg/kg, i.p) and after careful dissection of the right kidney; the right renal artery was cannulated via the mesenteric artery without interrupting the blood flow as described by Bowman (1970) [11]. The perfusate consisted of a modified Krebs–Henseleit solution (MKHS) of the following composition (in mMol/L): 114.00 NaCl, 4.96 KCl, 1.24 KH_2_PO_4_, 0.5 MgSO_4_.7H_2_O, 2.10 CaCl_2_ and 24.99 NaHCO_3_. Bovine serum albumin (BSA 6 g%; fraction V), urea (0.075 g), inulin (0.075 g) and glucose (0.15 g) were added to the solution, resulting in a final perfusate volume of 100 mL. The pH was adjusted to 7.4. In each experiment, 100 mL of MKHS were recirculated for 120 min. The pressure perfusion (PP) was measured at the tip of the stainless steel cannula in the renal artery. Samples of urine and perfused were collected at 10 min for analysis of the sodium, potassium and chloride levels by ion-selective electrodes (Rapid chen 744, Bayer Diagnostic, UK); inulin, as described by Walser et al. (1955) and modified by Fonteles et al. (1983); and osmolality, which was measured in vapor pressure osmometer (Wescor 5100C, USA). LAAO-*Bl* (10 μg/mL) was added to the system 30 min after the beginning of each perfusion. The perfusion pressure (PP), renal vascular resistance (RVR), urinary flow (UF), glomerular filtration rate (GFR), the percentage of sodium (% TNa^+^), potassium (%TK^+^) and chloride (%TCl) tubular transport were determined (Martinez-Maldonado and Opava-Stitzer, 1978). The results were compared to the internal control group at 30 min previously in each experiment (n = 6). This study was approved by the Ethics Committee on Animal Research (CEPA) of the Federal University of Ceara (Permit Number: 79/08). All efforts were made to minimize suffering.

### Statistical Analysis

The values given are mean ± S.E.M of triplicate values for each experiment. The significance of difference between the experimental groups and controls was accessed by a one-way analysis of variance (ANOVA). Significance was defined as p<0.05.

## Results

### LAAO-*Bl* induces apoptosis and necrosis in both renal epithelial cell lines

**Fig 1:**
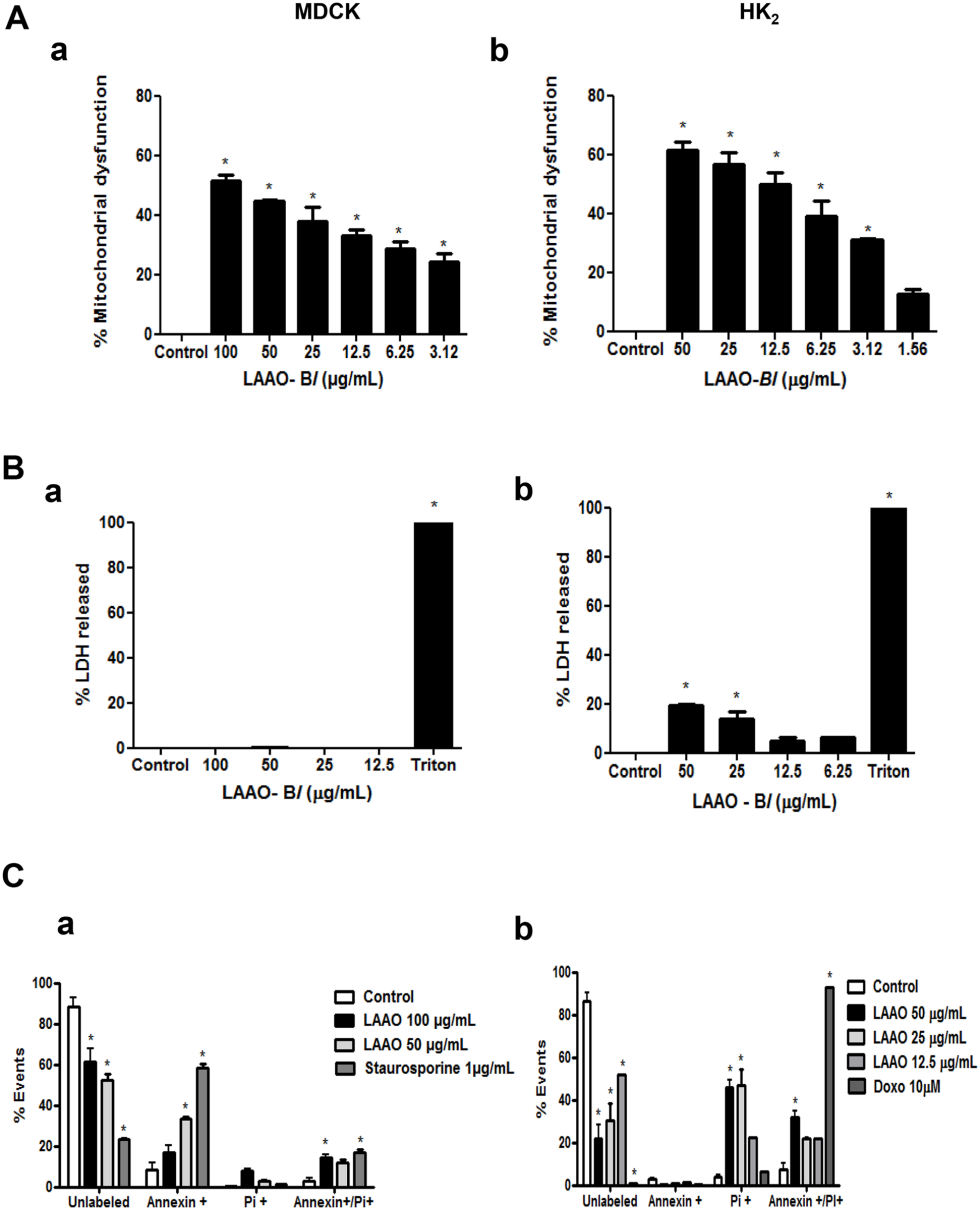
LAAO-*Bl* induces necrosis and apoptosis in renal epithelial cell lines. (A) MDCK (a) and HK-2 (b) cells were treated with LAAO-*Bl* (1.56–50/100 μg/mL) for 12 h. After treatment, cell viability was determined by MTT. (B) Percentage release of the lactate dehydrogenase enzyme of MDCK (a) and HK-2 (b) cells treated with the indicated concentrations of LAAO-*Bl*. Triton (7%) was used as positive control. (C) Annexin V^+^/PI^-^, Annexin V^+^/PI^+^ and AnnexinV^-^/PI^+^-populations were analyzed by flow cytometry in MDCK (a) and HK-2 (b) cells for 12 h after treatment with LAAO-*Bl*. Staurosporine (1 μg/mL) and doxorubicin (10 μM) for 12h were used as positive controls. All data are representative of three independent experiments in triplicate and are expressed as mean ± S.E.M. *Significantly different from control group (p<0.05, one way ANOVA, Dunnett post-test).

Previous studies reported that *Bothrops leucurus* venom was able to induce nephrotoxicity in the isolated perfused kidney from rats and was cytotoxic against renal tubular epithelial cells [2]. However, the cytotoxicity of LAAO-*Bl*, a major component of *B*. *leucurus* venom is poorly understood and studied. In order to clarify the mechanisms of nephrotoxicity and to compare whether LAAO-*Bl* has the same effects observed previously with total venom [2], cell death assays were conducted using two renal tubular cell lines such as MDCK and HK-2. In these cells treated with LAAO-*Bl*, 1.56–100 μg/mL for 12 h, there was a decrease in their viability in a concentration-dependent manner (Fig 1A, a-b). We next evaluated if necrosis was implicated in the cellular viability decrease observed by analyzing lactate dehydrogenase (LDH) release. Interestingly, in MDCK cells LDH release was not observed after 12 h of LAAO-*Bl* exposure (Fig 1B, a) while LAAO-*Bl* induced an apparent membrane rupture in HK-2 cells at the highest concentrations studied (50 and 25 μg/mL) when compared with untreated cells (Fig 1B, b). Aiming to identify the cell death modalities that were involved in our results we analyzed different markers of apoptosis and necrosis. The exposure of phosphatidylserine (PS) residues on the outer leaflet of the plasma membrane is usually considered a hallmark of apoptosis. Thus, cell staining with FITC-labeled Annexin V, which binds to PS, is considered a marker of early apoptotic events. Propidium iodide (PI) is a DNA intercalating agent that can be incorporated into cells only after major cell membrane damages (PI^+^). Hence, the combination of AnnV and PI cell staining can be used to distinguish apoptotic and necrotic cell death. Thus, annexin V/PI staining was applied to detect apoptotic/necrotic cells after LAAO-*Bl* treatment. In MDCK cells, LAAO-*Bl* significantly increased the percentage of early apoptotic (Annexin-V^+^, PI^-^), necrotic (Annexin-V^-^, PI^+^) and secondary necrotic cells (Annexin-V^+^, PI^+^) when compared with control untreated cells (Fig 1C, a). In HK-2 cells, in accordance with data obtained in the LDH-release assay, the Annexin-V-PI loading cell analysis demonstrated an increase in necrotic (PI^+^ cells) and secondary necrotic cells (Annexin-V^+^, PI^+^) in a concentration-dependent manner (Fig 1C, b). Treatment with staurosporine (1 μg/mL) and doxorubicin (10 μM) for 12h were used as positive controls.

### LAAO-*Bl* exposure induces mitochondrial hyperpolarization in MDCK cells

**Fig 2:**
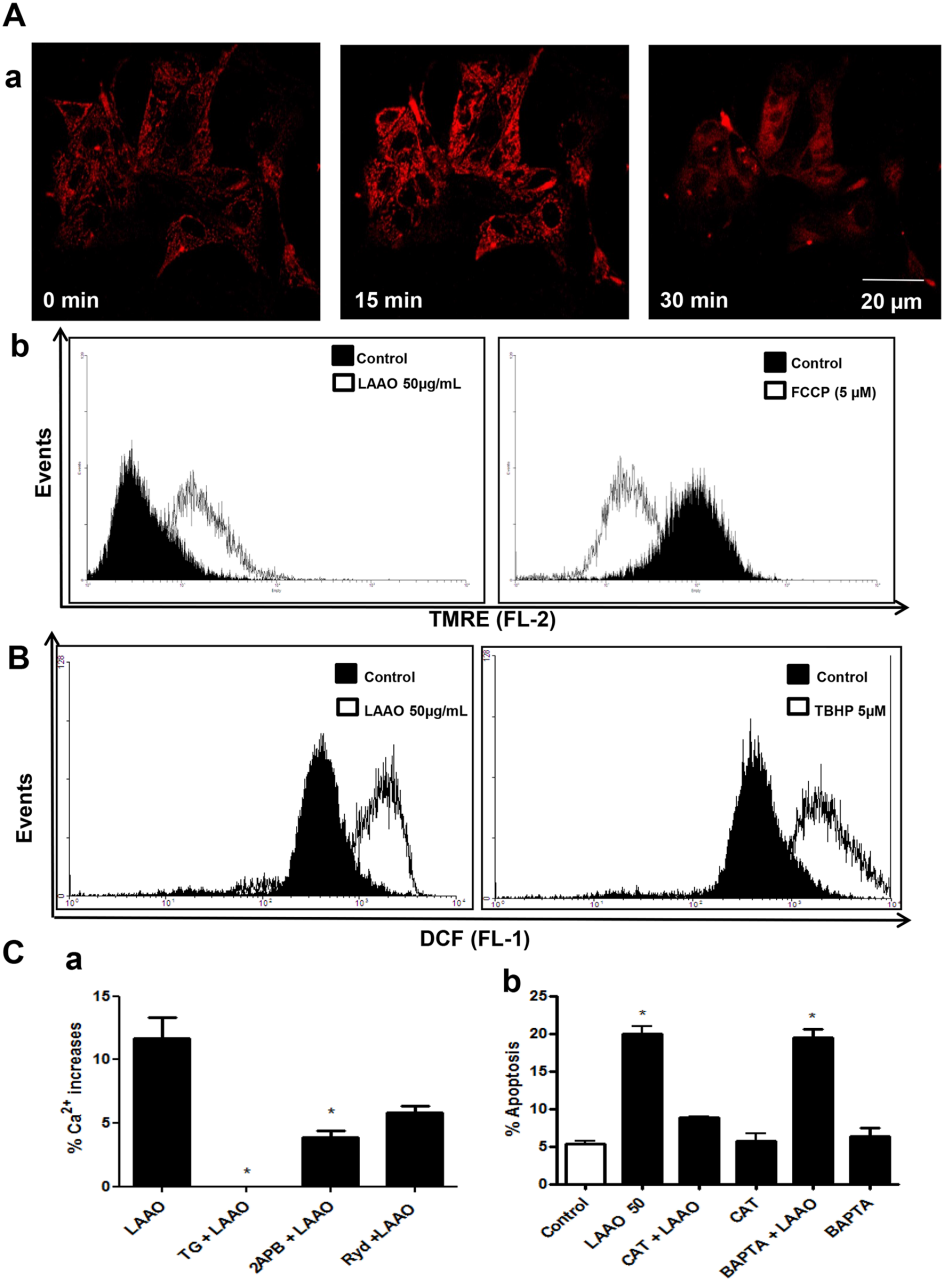
LAAO-*Bl* exposure induces mitochondrial hyperpolarization, intracellular Ca^2+^ mobilization and ROS generation in MDCK cells. (A) Changes on ΔΨm after LAAO-*Bl* (50 μg/mL) stimulation were measured by TMRE fluorescence (25 nM, 15 min at 37°C) and detected by confocal microscopy or flow cytometry. (a) Confocal images from cells TMRE loaded were collected at baseline and seconds after exposure with LAAO-*Bl* showing the increase of ΔΨm in MDCK cells in relation to basal levels. (b) Representative histograms obtained from MDCK cells TMRE loaded and exposed with LAAO-*Bl* (50 μg/mL). The uncoupler FCCP (5 μM) was used as positive control (b). (B) Intracellular LAAO-*Bl* (50 μg/mL) induced ROS generation in MDCK cells. After treatment for 12 h, cells were incubated with DCFH-DA (5 μM, 30 min at 37°C) then examined by flow cytometer. ROS generation was expressed as a ratio of relative fluorescent intensity compared to the control group. *t*-BHP (5 μM) was used as positive control. (C) (a) Cytosolic Ca^2+^ increase (%) induced by LAAO-*Bl* (50 μg/mL) was monitored in a real-time fluorescence microscope. Relative increase in maximum cytosolic Ca^2+^ response (%) evoked by LAAO-*Bl* (50 μg/mL) or after pre-incubation with TG (2 μM, 20 min) (TG + LAAO-*Bl*) or 2-APB (100 μM, 10 min) (2-APB + LAAO-*Bl*) or with Ryanodine (Ryd., 20 μM, 20 min) (Ryd. + LAAO-*Bl*). (b) The summary data quantifies the flow cytometry analysis of cells loaded with propidium iodide (PI, 50 μg/mL) under treatment with LAAO-*Bl* (50 μg/mL) for 12 hrs, in the presence or absence of catalase (100 μg/mL) or BAPTA-AM (20 μM). The data represent the mean ± S.E.M of at least three independent experiments. *Significantly different from control group (p<0.05, one way ANOVA, Dunnett post-test).

To verify the LAAO-*Bl* effects on mitochondrial transmembrane potential (**Δ**Ψm) cells were stained with the mitochondrial specific probe, tetramethylrhodamine ethyl ester (TMRE) and analyzed in a real-time confocal microcopy experiment and by flow cytometry. The images of the TMRE fluorescence changes observed in MDCK cells show a clear increase in fluorescence due to mitochondria hyperpolarization upon exposure of these cells to LAAO-*Bl* (Fig 2A, a). The increase of TMRE fluorescence was also observed in flow cytometry experiments (Fig 2A, b). The FCCP, nonspecific protonophore and uncoupler of oxidative phosphorylation, was used as a control. As expected, upon addition of FCCP (5 μM), TMRE fluorescence decreased immediately indicating mitochondrial depolarization.

### Cytosolic calcium and ROS generation involvement on the cytotoxicity and death induced by LAAO-*Bl* to renal epithelial cell

To determine the possible involvement of ROS production in the mechanism of action of LAAO-*Bl*. The ROS levels upon LAAO-*Bl* exposure were analyzed. For this purpose cells were stained with the 7′-Dichlorofluorescin diacetate (DCFH-DA) a cell-permeable non-fluorescent probe that turns to highly fluorescent 2′,7′-dichlorofluorescein upon oxidation. Fluorescence changes were analyzed using flow cytometry. LAAO-*Bl* was able to induce an increase of intracellular ROS levels as showed by the significant shift of the peak (shift in relative fluorescence intensity) when compared with untreated control group (Fig 2B). The oxidant agent tertButyl hydroperoxide-(t-BHP) was included as positive control. The participation of ROS on LAAO-*Bl*-mediated apoptosis was also evaluated in cells pre-treated with catalase (100 μg/mL, 30 min), a H_2_O_2_ scavenger. There was a significantly reduction in the percentage of cell death induced by LAAO-*Bl* under this condition (Fig 2C, b), suggesting the importance of ROS production in apoptosis LAAO-*Bl*-induced.

We next evaluated the intracellular Ca^2+^ levels in response to LAAO-*Bl*. In cells loaded with the intracellular calcium indicator Fura-2-acetoxymethyl ester (Fura-2AM), LAAO-*Bl* evoked a cytosolic Ca^2+^ increase. As the endoplamastic reticulum (ER) is the mainly source for cytosolic calcium release, the same study was done in cells treated before with thapsigargin (TG, 2 μM). Notably, the cytosolic Ca^2+^ mobilization by LAAO-*Bl* was decreased in this situation due to the inhibition of ER Ca^2+^ pump, SERCA. To define the mechanism which LAAO-*Bl* mediates Ca^2+^ release from ER, elucidating the possible involvement of channels in these responses, MDCK cells were pre-treated with different ER Ca^2+^ channel inhibitors, such as ryanodine (Ryd, 20 μM, 20 min) and 2-APB (100 μM, 10 min), that inhibit the Ca^2+^ release from RyR and IP_3_R, respectively. Our results showed that Ryd and 2-APB were able to decrease the Ca^2+^ rises LAAO-*Bl*-induced, suggesting a prominent regulation of these channels in LAAO-*Bl*-mediated Ca^2+^ increases (Fig 2C, a). To further identify the role of Ca^2+^ on apoptosis LAAO-*Bl*-mediated, we pre-treated the cells with BAPTA-AM (20 μM, 30 min), a Ca^2+^ chelator. Our data showed that apoptosis induced by LAAO-*Bl* in MDCK cells was not dependent on cytosolic Ca^2+^ release (Fig 2C, b).

### LAAO-*Bl* activates Bax, caspases 3 and 7 in epithelial kidney cells

**Fig 3:**
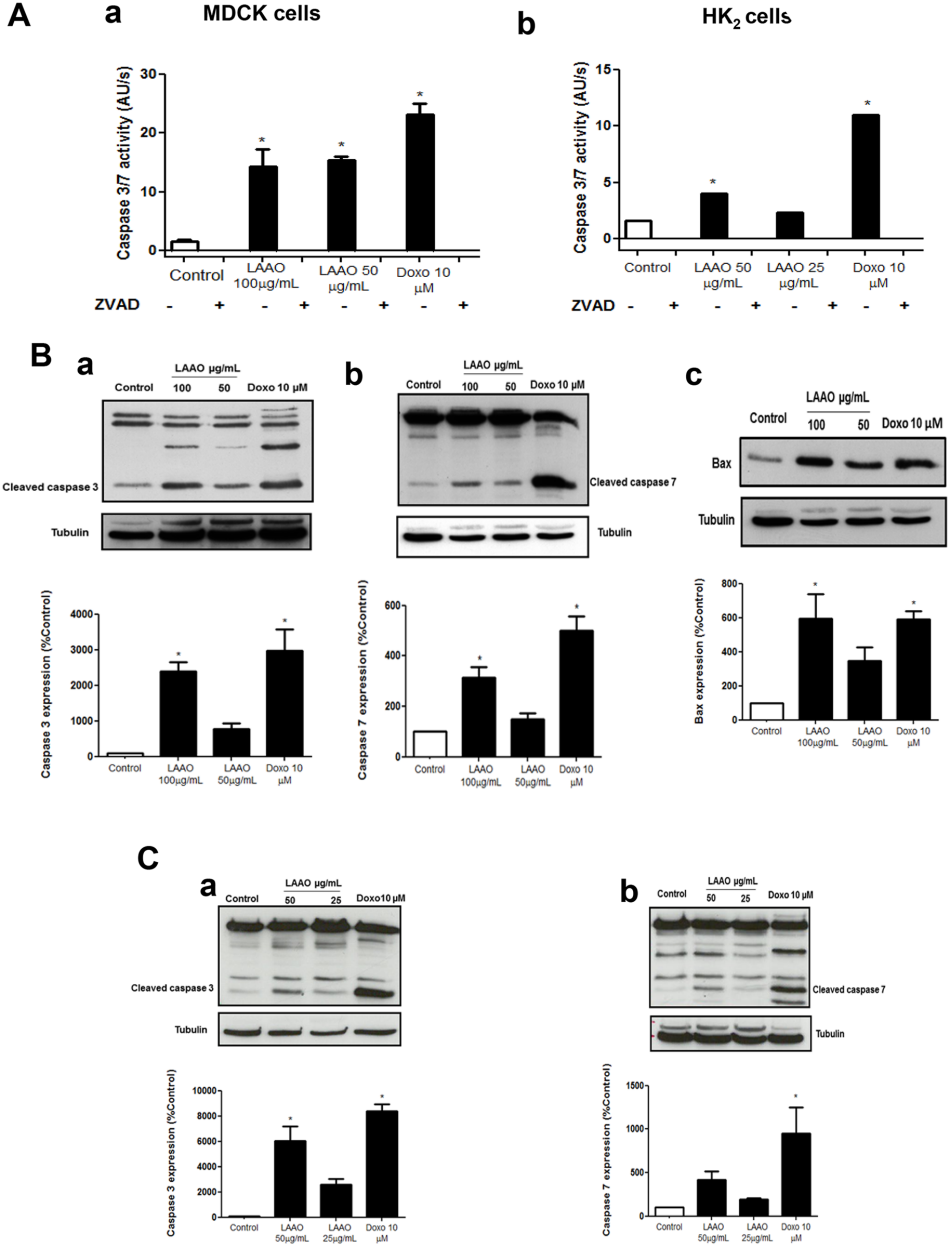
LAAO-*Bl* activates Bax, caspases 3 and 7 in epithelial kidney cells. (A) Caspase-3/7 activity was determined in the presence of the fluorogenic Ac-DEVD-afc substrate after treatment with LAAO-*Bl* (12 h) in the presence or absence of zVAD (10 μM) in MDCK (a) and HK-2 cells (b). (B-C) Western blot analyses were performed to detect the effects of LAAO-*Bl* on changes in Caspase-3 (a), -7 (b) and Bax (c) expressions from total protein of MDCK (B) and Caspase-3 (a), -7 (b) HK-2 (C) cell extracts. Percentage of Caspase-3 (a), -7 (b) and Bax (c) expression in MDCK (B) and caspase-3 (a) and caspase-7 (b) HK2 (C) treated with LAAO-*Bl* for 12 h. Data normalized in relation to Control group. Protein bands were analyzed and normalized to the densitometric values of α-tubulin. Doxorubicin was used as positive control (10 μM). Data are representative results of three independent experiments.

Our findings showed that LAAO-*Bl* induced a significant increase in the activities of caspase-3 or 7 in both cell lines (MDCK or HK-2), which were inhibited by pre-treatment with zVAD (10 μM, 30 min), respectively (Fig 3A a-b). However, this inhibition was unable to protect cells (Data not shown). Furthermore, LAAO-*Bl* induced the upregulation of cleaved caspase-3, caspase- 7 and the pro-apoptotic protein Bax after 12 h of exposure in a concentration-dependent manner (Fig 3B, a-c) in MDCK or in HK-2 cells (Fig 3C, a-b). Doxorubicin (Dox) 10 μM was used as a positive control.

### LAAO-*Bl* induced alterations in rat isolated kidney

**Fig 4:**
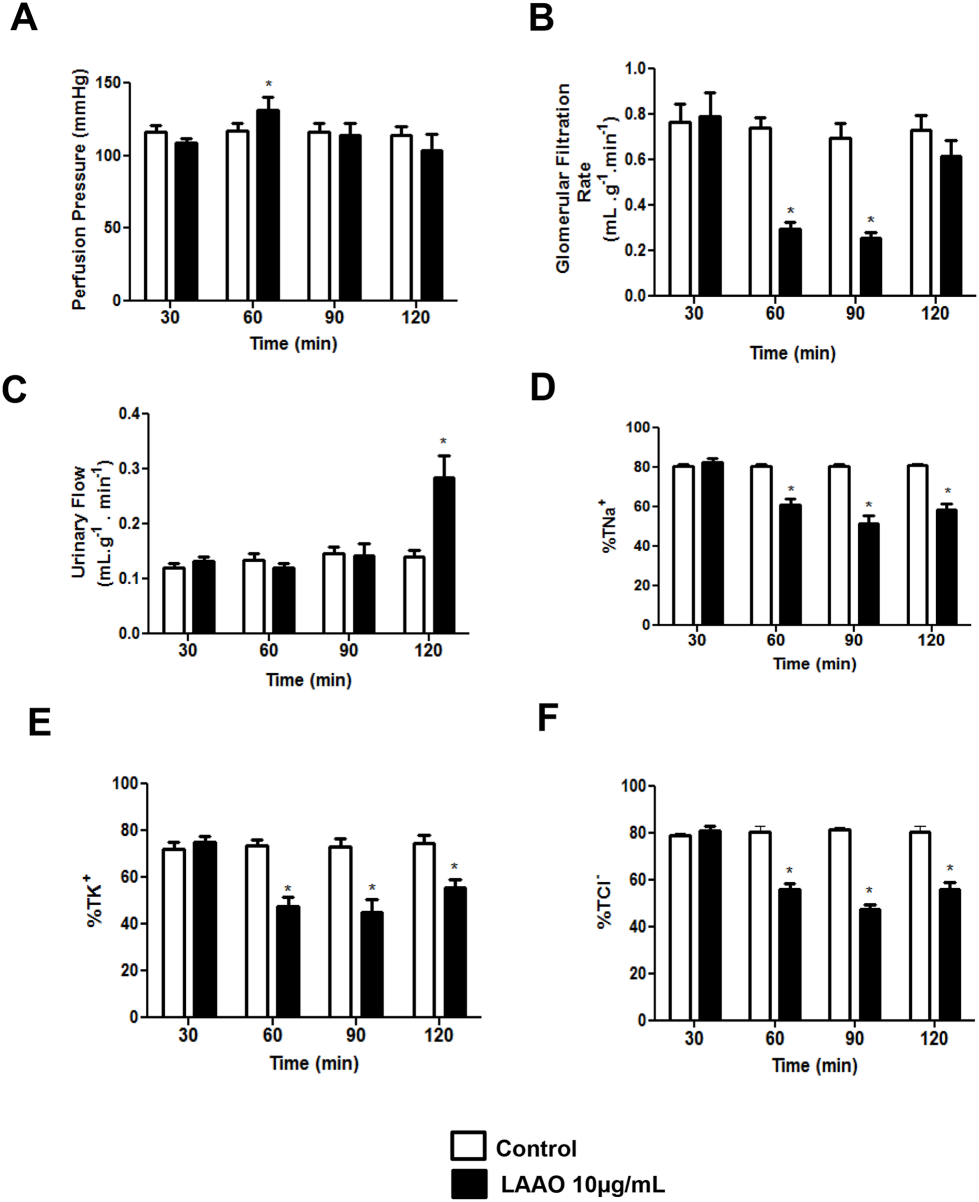
LAAO-*Bl* induced alterations in rat isolated kidney. Effects of LAAO isolated of *Bothrops leucurus* venom on perfusion pressure (A), glomerular filtration rate (B), urinary flow (C), sodium, potassiun and chloride (D, E, F) tubular transport percentage. LAAO-*Bl* was added to the system 30 min after the beginning of each perfusion. The data represent the mean ± S.E.M of at least six independent experiments. *Significantly different from control group (p<0.05, one way ANOVA, Bonferroni post-test).

Consistently with previous data, we used this experimental model in order to clarify the involvement of the LAAO-*Bl* in the renal effects of *Bothrops leucurus* venom. LAAO*-Bl* (10 μg/mL) increased perfusion pressure at 60 min and returned to normal at 90 min of perfusion (Fig 4A). There were no significant differences in the renal vascular resistance (RVR) after the infusion of LAAO-*Bl* when compared with control group. The glomerular filtration rate (GFR) decreased at 60 and 90 min of infusion and normalized at 120 min (Fig 4B). Urinary flow (UF) increased in the period of 120 min (Fig 4C). LAAO*-Bl* reduced significantly sodium, potassium and chloride tubular transport in the periods of 60, 90 and 120 min (Fig 4D–4F).

## Discussion

Acute renal failure is a common complication of *Bothrops* viper envenomation. High venom concentration at the renal tissue, direct action of venom components on renal tubules and renal epithelial cells, hemoglobinuria, vascular occlusion and oxidative stress are among factors incriminated in the pathogenesis of renal failure [12].

In order to understand the molecular mechanisms involved in the LAAO-*Bl* nephrotoxicity, we evaluated its cytotoxic to two renal tubule cell lines, HK-2, isolated from human tubule proximal [13] and MDCK, isolated from canine tubule distal [14]. Using Annexin/PI assay, it was observed that LAAO-*Bl* induced death in both cell lines, probably by different mechanisms. This result is in accordance with the literature showing possible involvement of apoptosis and necrosis in LAAO different cell types response [5, 15, 16, 17, 18, 19].

The tubular epithelial cells are the main targets of acute injury kidney for venoms [20]. Apoptosis, usually responsible for loss of kidney function, could be induced by many factors, including mitochondrial dysfunction, oxidative stress, Ca^2+^ imbalance and energy deprivation [13]. Some studies showed that mitochondrial dysfunction play an important role in renal cell damage by snake venoms [21, 2]. Here, we showed that LAAO-*Bl* (50 μg/mL) induced mitochondrial hyperpolarization in MDCK cells accompanied by ROS generation. In addition, some investigations suggested that **Δ**Ψm increases after induction of apoptosis, initiated by, e.g., ROS, Ca^2+^, Bax and ceramides [22, 23].

Since the apoptosis induced by LAAO is mainly attributed with a H_2_O_2_ generation [24, 25, 26, 27, 28, 29]. Here, intracellular H_2_O_2_ and ROS increase was monitored with DCFH-DA after stimulation with LAAO-*Bl* (50 μg/mL) in MDCK cells. Furthermore, H_2_O_2_ removal by catalase decreased, approximately, 50% of the apoptotic effects, suggesting that H_2_O_2_ plays a main role to induce apoptosis LAAO-*Bl*-mediated. Interestingly, the cytosolic Ca^2+^ rises mediated by LAAO-*Bl* (50 μg/mL) were from endoplasmic reticulum (ER) via IP_3_ receptors, since ROS and oxidative stress disrupt the ER function, inducing Ca^2+^ rises [30]. Although Ca^2+^ participates of cell damage, BAPTA-AM was not able to reduce apoptosis LAAO-*Bl*-regulated.

Two major apoptotic signaling pathways, including the intrinsic and extrinsic signals, converge onto caspases to initiate cell death [31]. In this study, we demonstrated that LAAO-*Bl* triggered the activation of caspase 3 and 7 in MDCK and HK-2 cells. Furthermore, we showed that LAAO treatment up-regulated the expression of pro-apoptotic protein Bax. Other reports showed that LAAO induced apoptosis through caspase-dependent [5, 24, 32, 33].

To verify the effect of LAAO-*Bl* in the kidney without interference of systemic factors, we used perfusion in the rat kidney. LAAO- *Bl* (10 μg/mL) showed an increase in perfusion pressure and urine flow, with no effect in renal vascular resistance. It was observed a decrease in glomerular filtration rate. The tubular transport of sodium, potassium and chloride was reduced significantly. The loss of tubular epithelial cells function induced for LAAO- *Bl* can explain some of the renal effects observed in this study, such as increased of the urine flow and reducing the transport of electrolytes. Using the same model, we showed that *B*. *leucurus* venom also reduced the GFR, but decreased the PP and UF. This venom also reduced the sodium and potassium tubular transport [2].

Among several enzymes in *Bothrops* venom, L-amino acid oxidase, phospholipase A_2_, metalloprotease and sphingomyelinase are believed to contribute to renal toxicity [34]. In addition, perfusion of isolated rat kidneys with LAAO [35], C type lectins [36, 37], phospholipase A2 (PLA2) myotoxins [38, 39] and thrombin-like enzyme [40] from *Bothrops* venoms caused renal function disturbances.

In conclusion, we showed that LAAO-*Bl* induced intense alterations in renal physiology, assessed in the isolated rat kidney. These direct nephrotoxicity effects are in agreement with loss of tubular epithelial cell function, as it has been demonstrated that LAAO-*Bl* induced apoptosis in tubular epithelial cells by oxidative stress.

Our results have important implications for understanding the mechanisms of nephrotoxicity triggered by this family of enzymes. Since these molecular events are responsible for acute kidney injury caused by ophidism, it contributes to develop drugs and more effective therapeutic strategies.
